# Metaproteomics insights into fermented fish and vegetable products and associated microbes

**DOI:** 10.1016/j.fochms.2021.100045

**Published:** 2021-10-22

**Authors:** Emmanuel Sunday Okeke, Richard Ekeng Ita, Egong John Egong, Lydia Etuk Udofia, Chiamaka Linda Mgbechidinma, Otobong Donald Akan

**Affiliations:** a Department of Biochemistry, Faculty of Biological Sciences, University of Nigeria, Nsukka 410001, Enugu State, Nigeria; b Natural Science Unit, School of General Studies, University of Nigeria, Nsukka 410001, Enugu State, Nigeria; c Environmental Science and Engineering, School of Environmental Science and Safety Engineering, Jiangsu University, Zhenjiang 212013, PR China; d Department of Biological Sciences, Ritman University, Ikot Ekpene, Akwa Ibom State, Nigeria; e Microbiology Department, Faculty of Biological Sciences, University of Nigeria, Nsukka, Nigeria; f Department of Zoology, Faculty of Biological Science, Akwa-Ibom State University, Ikot Akpaden, Uyo, Akwa-Ibom State, Nigeria; g Ocean College, Zhejiang University, Zhoushan 316021, Zhejiang, China; h Department of Microbiology, University of Ibadan, Ibadan, Oyo State 200243, Nigeria; i College of Food Science and Engineering, Central South University of Forestry and Technology, Changsha, Hunan 41004, China; j Microbiology Department, Faculty of Biological Science, Akwa-Ibom State University, Ikot Akpaden, Uyo, Akwa-Ibom State, Nigeria

**Keywords:** Proteomic analysis, Fermented fish, Fermented vegetables, Fermentation, Fermentative microbes, Mass spectroscopy

## Abstract

The interest in proteomic studies of fermented food is increasing; the role of proteins derived from fermentation extends beyond preservation, they also improve the organoleptic, anti-pathogenic, anti-cancer, anti-obesogenic properties, and other health conferring properties of fermented food. Traditional fermentation processes are still in use in certain cultures, but recently, the controlled process is gaining wider acceptance due to consistency and predictability. Scientists use modern biotechnological approaches to evaluate reactions and component yields from fermentation processes. Pieces of literature on fermented fish and vegetable end-products are scanty (compared to milk and meat), even though fish and vegetables are considered health conferring diets with high nutritional contents. Evaluations of peptides from fermented fish and vegetables show they have anti-obesity, anti-oxidative, anti-inflammatory, anti-pathogenic, anti-anti-nutrient, improves digestibility, taste, nutrient content, texture, aroma properties, etc. Despite challenges impeding the wider applications of the metaproteomic analysis of fermented fish and vegetables, their potential benefits cannot be underestimated.

## Highlights

• Increasing global population means higher demand for healthy food.
• Fish and vegetables are healthy foods, but overproduction leads to spoilage.
• Fermentation of fish/vegetables elongate their shelf lives, improved flavour and functions.
• Microbes associated with Fish/vegetable fermentation produce health conferring peptides.
• There is little review on peptides elicited during fish/vegetable fermentations.

## 1 Introduction

One of the main pivots of human health is the type and quantity of diet they consume. Fish and vegetables are known health-conferring sources of food (Olovo et al., 2021, Méndez and Pazos, 2017); however, due to their high nutritional contents and water activity, they are easily degraded, thus lowering their shelf-life and potentially becoming vectors for pathogenic microorganisms. As the world’s population burgeons toward the 9.6 billion projected mark by 2050, the demand for food would also increase. To sustain the huge food demand and keep populations healthy, there must be sufficient healthy food supplies (Chaudhary, Bhalla, Patiyal, Raghava, & Sahni, 2021). The overproduction of food to meet huge demands could also lead to spoilage, especially in places without proper storage facilities. Microbes inherent in foods are versatile; some spoil foods, while others help preserve raw foods through fermentation processes, given suitable environmental conditions. Microbes self-perpetuate while interacting with food molecules. During fermentation, they elongate the end-products shelf life, improve their nutritional contents, aroma, taste, and texture relative to the raw food material (s) (Voidarou et al., 2021, Marco et al., 2017). According to Voidarou et al. (2021), Louis Pasteur defined fermentation as ‘life without oxygen’- a process that yields end-products like CO_2_, ethanol, organic acid, and other organic molecules: important health and industrial products.

Fermentation processes can either be controlled (novel, modern, or industrial, with active allochthonous microbes) or uncontrolled (traditional, natural, or spontaneous, with active autochthonous microbes) and result in the biotransformation of raw food components (Marco et al., 2017). The numerous possible end-products, their bioactivities, and food security seem to be driving forces for the widening applications of fermentation processes in modern times. Functional microbes produce peptides that improve the organoleptic, preservative, digestive, anti-oxidative, probiotic, anti-microbial, anti-toxin, and anti-anti-nutrient outcomes in fermented foods (Voidarou et al., 2021, Sharma et al., 2020); these attributes make fermented end-products good natural therapeutics, and can be utilized to curb westernized diets-induced disease conditions like obesity and cardiovascular diseases.

It is common knowledge that consumers of fermented products consume ‘live microbes’ and transformed molecules together; and that these components confer health (Voidarou et al., 2021, Marco et al., 2017). Fermentation has surpassed the ‘preservation’ narrative; therefore, it is imperative to study these molecules, elucidate their characteristics, interactions, and find possible applications, especially in the formulation of good foods. Proteomics biochemically measures low molecular weight compounds like peptides, amino acids, aldehydes, organic acids, and amines (Wang, Xia, Gao, Xu, & Jiang, 2017), helps monitor certain of these molecules as fermented food’s safety and quality biomarkers (Méndez & Pazos, 2017). There are numerous metaproteomic studies on fermented dairy and certain meat products, but works on fermented fish and vegetable products are scanty. Fish (unsaturated fatty acids like omega fatty acids) and vegetables (bioactive substances like polyphenols) are known for their high nutritional contents (Olovo et al., 2021, Méndez and Pazos, 2017), fermentation can yield even healthier end-products. In this review, we outlined modern proteomic methods and their usefulness; reviewed literature works on fermented fish and vegetable peptides; microbes associated with fish and vegetable fermentations; and challenges encountered in proteomic studies.

## 2 Metaproteomic analysis methods

Studies on fermented food proteins- metaproteomic analysis allow for the accurate identification and quantification of proteins; and provides details on their authenticity, origin, biological activities, allergenicity, and sensory properties. Scientists use information gotten from such studies either for descriptive or/and comparison purposes: descriptive analysis provides a crucial understanding of metabolic activities of the microbial communities under specific conditions (Heyer et al., 2019), while comparative analysis simultaneously elucidates the taxonomic composition and functionality of microbial communities in different fermentation processes, and micro-ecology (Heyer et al., 2019). The characterization of proteins occurs at genomic, transcriptomic, and post-transcriptional levels, while their metaproteomic profiles fall under quality, quantity, and functions categories (Ji et al., 2017).

The quantitative approach provides the relative abundance information of a specific protein while focusing on identifying and characterizing the complete protein set present in the fermented food, including their post-translational modifications (PTMs). On the other hand, the functional approach deals with the functional interaction(s) between proteins or between a protein and other molecules. The qualitative properties of proteins are retrieved from either a non-assembled metagenome database, an assembled metagenome database, or a built taxonomy database (Geron, Werner, Wattiez, Lebaron, & Matallana-Surget, 2019).

**Fig. 1:**
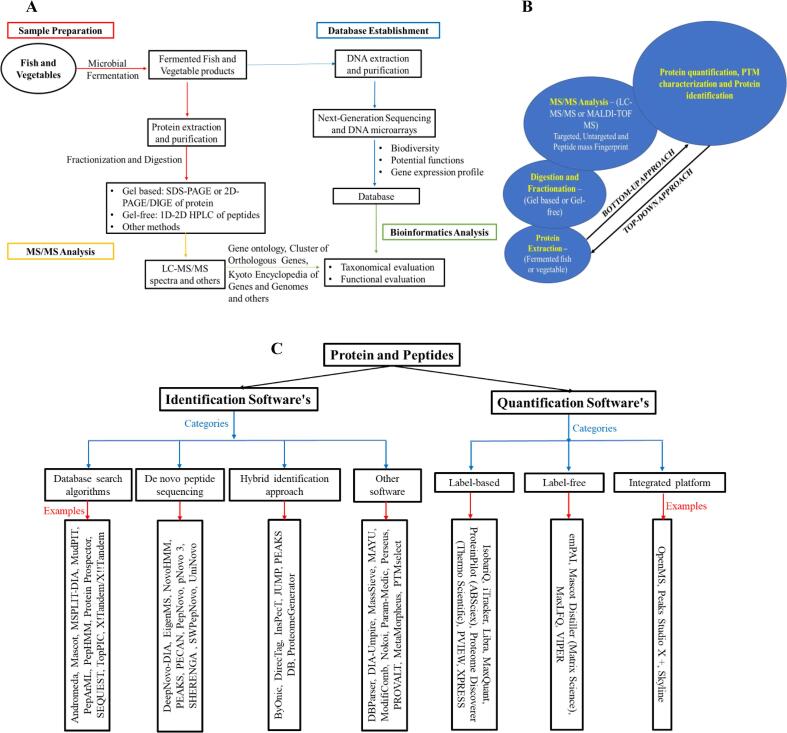
Metaproteomic Studies of Fermented Food Peptides. Fig. 1**A:** The schematic workflows for the metaproteomics of fermented fish and vegetables. Fig. 1**B:** illustrates the bottom-up and top-down MS-based metaproteomics approaches commonly used in fermented fish and vegetable research; Fig. 1**C:** showcases the common software tools used for peptide and protein identification and quantification.

The workflow for the metaproteomic analysis of fermented fish and vegetables is as illustrated in Fig. 1a. It shows protein extraction from fermented foods and subsequent purification processes. Proteins digested into fragmented peptides are then analyzed with different high-performance separation techniques such as one-dimension and multi-dimension chromatography, two-dimension gel electrophoresis, and high-resolution mass spectrometry. These methods monitor protein compositions in fermented foods and the changes during the fermentation process (Ortea, O’Connor, & Maquet, 2016). Proteomic studies reveal processes that identified proteins go through and can be a useful tool for toxin, allergen, nutritional value, and storage authentications.

### 2.1 Total protein extraction method

Total protein extraction from fermented foods of interest is the first key step in metaproteomic studies. Factors like differences in cell wall structures and inherent microbial cell lysis resistance level affect protein extraction (Zhang et al., 2018). For fish and vegetable products, another important factor aside from the characteristics of the microbial community is the heterogeneity in salinity and alkalinity of fish and vegetables, respectively (Liu et al., 2021a). These microbial and food factors affect the kind and quality of the extractable proteins to varying degrees.

The basic extraction protocol for proteins involves the physical disruption of cells, dissolution with buffer, purification by precipitation, and protein lysis. The buffer, precipitant, and lysate choices should be considered carefully as they are important for the release of proteins from fermenting microbes (Hayoun et al., 2019). Detergents such as sodium dodecyl sulfate (SDS) and 3-(3-Cholamidopropyl) dimethylammonium)-1-propane sulfonate (CHAPS) show high efficiencies with protein lysis and improvements can be achieved when incorporated with grinding, bead beating, and ultrasonication (Kunath et al., 2019). However, important factors like substrates, products, and repeatability should be considered when choosing methods.

### 2.2 Separation/purification of extracted proteins

The separation and purification of peptides follow after the extraction process, and several methods can be employed just before analyzing the peptides with mass spectrometers. Some of the methods are:

#### 2.2.1 Gel-based separation

Gel separation is one of the commonest methods used for protein separation, uses a 2D polyacrylamide gel electrophoresis/differential in-gel electrophoresis (2D-PAGE/DIGE), and yields about 2000 discrete protein spots after staining (Anguraj Vadivel, 2015). Protein expression herein is a factor of the spot size and intensity. Gel separations are done either in single (1D), two (2D), or three (3D) dimensions. The single dimension method of Gel separation bases on the protein’s molecular weight (using sodium dodecyl sulfate–polyacrylamide gel electrophoresis SDS-PAGE). The two-dimension method bases on the protein’s isoelectric point (using immobilized pH gradient gel strips) and molecular weight (SDS-PAGE). Although the 1D and 2D methods are common, their inability to detect post-translational modifications (as with fish and vegetable proteins) (Arya, Prakash, Sougrakpam, & Deswal, 2021) is a major setback.

Colignon, Raes, Dieu, Delaive, and Mauro (2013) adverted the setback by developing the three-dimension method. The 3D uses isoelectric focusing and sample fractionation followed by two consecutive separations with SDS-PAGE, with two different buffer systems. The 3D separation method evades the co-migration interferences that affect protein resolution while providing a wide range of applications in quantitative profiling of complex proteomes and identifying post-translational modification (Colignon et al., 2013). In addition, another method developed for easy peptide recovery uses a disulfide-containing analog of bis-acrylamide called bis-acrylylcystamine (BAC). Peptides released from the gel can be enhanced by adding tris-(2-carboxyethyl) phosphine (TCEP) for a complete dissolution with BAC-cross-linked acrylamide gel (Takemori, Takemori, Ishizaki, & Hasegawa, 2014). BAC gels can be used for improved complex membrane proteins recovery before mass spectroscopic analysis since low quantity proteins cannot be detected. Incorporating BAC gel with 2D and 3D separation methods, protein recovery, identification, and separation resolution from in-gel digestion is improved.

#### 2.2.2 Gel-free separation

Gel-free separation methods solve most of the limitations with the gel-based method and can also serve as a suitable complement depending on the fermented food type and target protein. The most common approach used is the multidimensional protein identification (MudPIT) with strong cation-exchange (SCX) fractionation, reversed-phase (RP) chromatography, and tandem mass spectrometer (MS/MS). MudPIT is an important tool in food proteomic researches and had been used in the study of pumpkin (*Cucurbita maxima*) and lettuce (*Lactuca sativa*) (Won *et al*., 2010). Another comparative approach is incorporating anion/cation exchange reversed-phase chromatography to chromatographically separate proteins (Quan, Feng, Lui, Shi, & Chu, 2017). This combination achieves a 2D separation. Wu, Li, Huang, and Zhang (2021) identified certain proteins with this method and reported that the method was efficient with a high degree of automation and accurate protein information.

#### 2.2.3 Other approach(es)

There are other recently reported methods with efficient protein extract digestion yield and reduced application time. Two examples of these methods are the suspension-trapping filter-based approach (S-Trap) and solid-phase-enhanced sample preparation (SP3). The SP3 method is more efficient in speed and peptide delivery (Hayoun et al., 2019). However, other methods such as molecular imprinting, microfluidic chip, magnetic separation, reverse micelles, and crystallization also have high precision and efficiency when combined with other separation technologies (Liu et al., 2020).

### 2.3 Mass spectroscopic (MS) detection and analysis

#### 2.3.1 MS detection

After the use of gel-based or gel-free methods to sufficiently reduce sample complexities, the peptides are mixed and analyzed using mass spectroscopy. In proteomic studies, the most used ionization methods are electrospray ionization (ESI) (Chen et al., 2017) and matrix-assisted laser desorption/ionization (MALDI) (Kailasa et al., 2020). However, electrospray ionization is employed more due to its high-throughput secondary spectra of peptides. Directly coupling liquid chromatography (LC) with ESI–MS achieves a highly automated detection of peptides in fermented food (Liu et al., 2021b, Ortea et al., 2016), whether targeted or non-targeted (Hart-Smith, Reis, Waterhouse, & Wilkins, 2017).

Targeted quantitative metaproteomics has gained popularity when used with mass spectrometry-based protein quantification because it can detect and analyze specific sets of proteins with high sensitivity, accuracy, and reproducibility (Song et al., 2017). According to Hart-Smith et al. (2017), targeted proteomic assays can be accomplished through the acquisition of peptide MS/MS data using selected reaction monitoring (SRM), parallel reaction monitoring (PRM), or targeted data acquisition (TDA). Also, a targeted proteomic assay can be achieved via comprehensive data-independent acquisition (DIA) strategies, e.g., sequential window acquisition of all theoretical spectra (SWATH), which produces extractable quantitative data using 10,000 proteins assay libraries (Sim et al., 2020).

Non-targeted quantitative proteomics has been explored more in foods. It is a more exploratory, data-dependent acquisition (DDA) using LC-MS/MS analysis. The highest abundance of peptide ions from full MS scans is selected for MS/MS (Yesiltas et al., 2021). Herein, highly abundant microbial proteins (>1% relative abundance by riBAQ) are selected for bioinformatic analysis, and the full-length sequences from microbial and food proteins were analyzed using EmulsiPred (https://github.com/MarcatiliLab/EmulsiPred). Other methods that can employ non-targeted approaches include isobaric tags for relative and absolute quantitation (iTRAQ), and tandem mass tags or label-free. These methods were recently employed for the protein profiling of certain food substances (like commercial soybean milk and quinoa seeds) (Galindo-Luján et al., 2021).

#### 2.3.2 MS data analysis

In proteomic studies, MS/MS is commonly used for protein identification compared with peptide mass fingerprinting. For fermented fish and vegetable proteins, the use of this procedure becomes important because there is limited information in the public database on related proteins. Fig. 1b shows the schematic illustration for the bottom-up and top-down MS-based metaproteomics approaches suitable for use in fermented fish and vegetable protein researches. With the top-down proteomics, intact or large protein fragments are directly subjected to gas-phase fragmentation for MS/MS analysis; by contrast, the bottom-up approach is widely used for protein identification by MS. Complex mixtures, or purified proteins are subjected to proteolytic cleavage; while MS or MS/MS is used to analyze resulting peptides. The commonly used software for peptide and protein identification and quantification are categorized based on their functions (Chen, Hou, Tanner, & Cheng, 2020), as presented in Fig. 1C. SwissProt, NCBInr, PIR, and EMBL are a few public databases tools; however, they do not include fermented fish and vegetable peptide data. The unavailability of fermented fish and vegetable protein data increases the risk of misidentifying newly extracted peptides from these fermented foods. Available protein databases are either generated from shotgun metagenomic data or are pseudo-metagenomics databases constructed from proteins obtained from a public database according to the microbial community structure obtained by 16S rRNA analysis (Muth, Renard, & Martens, 2016). Recently, transcriptome-derived protein databases are gaining increasing attention as manually curated food proteomics databases such as FermFooDb (https://webs.iiitd.edu.in/raghava/fermfoodb/) (Chaudhary et al., 2021). FermFooDb comprises 2205 entries with relevant fields like peptide sequence, Mass and IC50, food source, functional activity, fermentation conditions, starter culture, testing conditions of sequences *in vitro* or *in vivo*, type of model, and method of analysis.

### 2.4 Bioinformatics analysis

The bottom-up proteomic approach requires taxonomy-specific peptides for microbial community identification, quantification, functional annotation, metabolic, and biological classification. Thus, bioinformatics data is an integral part of metaproteomics (Krutz, 2019); some analytical tools used include the Protein ANalysis Through Evolutionary Relationships (PANTHER); Database for Annotation, Visualization and Integrated Discovery (DAVID); Ingenuity Pathway Analysis (IPA, QIAGENs Redwood City, www.qiagen.com/ingenuity); Gene Ontology (GO); Cluster of Orthologous Groups (COG); and Kyoto Encyclopedia of Genes and Genomes (KEGG) (http://geneontology.org/) (Liu et al., 2021b, Heyer et al., 2017). Also, the protein–protein interactions databases, like the Search Tool for the Retrieval of Interacting Genes/Proteins (STRING); Molecular INTeraction database (MINT); IntAct, Microbial Protein Interaction Database (MPIDB) as extensively described by Calderón-González, Hernández-Monge, Herrera-Aguirre, and Luna-Arias (2016) are also used. Among the commonly employed bioinformatics tools, the GO provides controlled, structured vocabularies and classifications that focus on the knowledge of gene products’ roles in cells. These gene products are classified either as cellular components (CC), biological processes (BP), and molecular functions (MF) (Galindo-Luján et al., 2021). The COG represents a phylogenetic classification of proteins from completely sequenced genomes used to distinguish evolutionary relationships between orthologs and paralogs (Heyer et al., 2017), while KEGG analytical tool groups microbial genes and proteins according to their molecular levels and functional information (L et al., 2021b).

## 3 Proteomic analysis of fish and vegetables

### 3.1 Proteomic analysis of fish

Fish consumption is growing rapidly at an average annual per capita fish consumption rate of 9.0–17.8 kg (FAO, SOFIA, 2014). The high consumption volume is due to extensive outdoor fish cultivation that meets high demands at very cheap rates. Fish is a cheap source of food and contains a high amount of nutrients such as proteins, vitamins, minerals, long-chain polyunsaturated fatty acids (PUFs), eicosapentaenoic (EPA), docosahexaenoic acid (DHA), peptides, selenium, and taurine; these nutrients help prevent and treat metabolic diseases (Méndez & Pazos, 2017). According to Jacek (2005), the high nutritional content and water activity of fish becomes a disadvantage for its shelf-life. In addition, fish muscle proteins are heat-labile, have an abundance of proteolytic enzymes, and aids the proliferation and survival of psychrophilic microbes. These factors combined lower fish’s shelf life when compared to terrestrial animals’ meat.

The shelf life of harvested fish can be extended either by salting, smoking, or drying; however, when left for extended periods, the activities of co-functioning multiple microorganisms and their enzymes ferment the minimally processed fish- yielding unique organoleptic properties and extending the fish product’s shelf-life (Ji et al., 2017). The microorganisms and their enzymes convert certain organic materials into simpler compounds associated with unique aromas, texture, and flavour characteristics found in fermented fish products. There are two classifications for fermented fish according to Zang, Xu, Xia, and Regenstein (2019) (a) by the nature of their end products; (i) whole or sliced, but the fish still retains its structure, (ii) paste, here the fish is fermented in paste form, and (iii) sauce, here the fish is fermented in a broth or liquid and (b) by the fermentation method adopted; (i) traditional or spontaneous, here natural or autochthonous microbes bio-transform the fish and (ii) starter culture, here cultured or allochthonous microbes bio-transform the fish.

Different organoleptic outcomes for fermented fish, depending on countries of origin, fish types, and environmental factors (process) (Waisundara, Jayawardena, & Watawana, 2016). Modern sciences like microbiology and biotechnology help elucidate microbial bio-transformations of raw or minimally processed food items like fish. The bacteria *Photobacterium profundum* was the most abundant identified microbe in fish fermentation studies conducted by Ji et al. (2017). Functional microbes involved in fermentative processes yield useful end-products during their primary and secondary metabolic activities; their actions on fish could either be acidification (or carbohydrate catabolism) -that yields anti-microbial peptides (extends shelf-life), gelation of myofibrillar and sarcoplasmic proteins of the muscles- peptides that change the elasticity, cohesion, and hardness of end-products, and the degradation of proteins and lipids that yields myriad of peptides that allude to flavourful, tasty compounds and easily digestible and absorbable nutrients (Zang et al., 2019). Table 1 shows peptides identified from fermented fish.

**Table 1:** List of Important Identified Peptides from Fish and Vegetable Fermentation Processes.

Fermented Fish Products				
Fish Product	Fermenting microbes	Identified proteins and peptides	Function	Reference
*Siniperca chuatsi*	*Photobacterium profundum*	Ornithine carbamoyltransferase, catabolic	Energy metabolism and amino acid metabolism	Ji et al. (2017).
*Siniperca chuatsi*	*Photobacterium profundum*	Phosphoenolpyruvate carboxykinase [ATP]	Energy metabolism and amino acid metabolism	Ji et al. (2017).
*Siniperca chuatsi*	*Vibrio parahaemolyticus*	Malate dehydrogenase	Energy metabolism and amino acid metabolism	Ji et al. (2017).
*Siniperca chuatsi*	*Photobacterium profundum*	Ornithine carbamoyltransferase, catabolic	Energy metabolism and amino acid metabolism	Ji et al. (2017).
*Siniperca chuatsi*	*Clostridium thermocellum*	Enolase	Energy metabolism and amino acid metabolism	Ji et al. (2017).
*Siniperca chuatsi*	*Photobacterium profundum*	Ornithine carbamoyltransferase, catabolic	Energy metabolism and amino acid metabolism	Ji et al. (2017).
*Siniperca chuatsi*	*Salmonella paratyphi*	Arginine deiminase	Energy metabolism and amino acid metabolism	Ji et al. (2017).
*Siniperca chuatsi*	*Photobacterium profundum*	Phosphoenolpyruvate carboxykinase [ATP]	Energy metabolism and amino acid metabolism	Ji et al. (2017).
*Siniperca chuatsi*	*Cupriavidus necator*	Triosephosphate isomerase	Energy metabolism and amino acid metabolism	Ji et al. (2017).
*Siniperca chuatsi*	*Xylella fastidiosa*	Ubiquinone/menaquinone biosynthesis C-methyltransferase	Energy metabolism and amino acid metabolism	Ji et al. (2017).
*Siniperca chuatsi*	*Acinetobacter baumannii*	Outer membrane protein Omp38	Energy metabolism and amino acid metabolism	Ji et al. (2017).
*Siniperca chuatsi*	*Vibrio tasmaniensis*	50S ribosomal protein L1	Genetic information processing and cellular processes	Ji et al. (2017).
*Siniperca chuatsi*	*Photobacterium profundum*	60 kDa chaperonin	Genetic information processing and cellular processes	Ji et al. (2017).
*Siniperca chuatsi*	*Shewanella violacea*	Cold shock-like protein CspA	Genetic information processing and cellular processes	Ji et al. (2017).
*Siniperca chuatsi*	*Shewanella violacea*	Cold shock-like protein CspA	Genetic information processing and cellular processes	Ji et al. (2017).
*Siniperca chuatsi*	*Pseudoalteromonas haloplanktis*	Elongation factor	Genetic information processing and cellular processes	Ji et al. (2017).
*Siniperca chuatsi*	*Pseudoalteromonas haloplanktis*	60 kDa chaperonin	Genetic information processing and cellular processes	Ji et al. (2017).
*Siniperca chuatsi*	*Vibrio campbellii*	DNA-directed RNA polymerase subunit beta	Genetic information processing and cellular processes	Ji et al. (2017).
*Siniperca chuatsi*	*Pseudoalteromonas haloplanktis*	Polyribonucleotide nucleotidyltransferase	Genetic information processing and cellular processes	Ji et al. (2017).
*Siniperca chuatsi*	*Vibrio anguillarum*	Flagellin	Genetic information processing and cellular processes	Ji et al. (2017).
*Siniperca chuatsi*	*Pseudoalteromonas haloplanktis*	60 kDa chaperonin	Genetic information processing and cellular processes	Ji et al. (2017).
*Siniperca chuatsi*	*Bifidobacterium adolescentis*	tRNA-2-methylthio-N (6)-dimethylallyladenosine synthase	Genetic information processing and cellular processes	Ji et al. (2017).
*Siniperca chuatsi*	*Vibrio cholerae*	DNA-directed RNA polymerase subunit alpha	Genetic information processing and cellular processes	Ji et al. (2017).
*Siniperca chuatsi*	*Acinetobacter baumannii*	Chaperone protein DnaK	Genetic information processing and cellular processes	Ji et al. (2017).
*Siniperca chuatsi*	*Acinetobacter baylyi*	60 kDa chaperonin	Genetic information processing and cellular processes	Ji et al. (2017).

Fermented Vegetable Products				
Vegetable Product	Fermenting microbes	Identified proteins and peptides	Function	Reference
Pickled radish	*Lactiplantibacillus plantarum*, *Lactobacillus pentosus*, *Limosilactobacillus fermentum*	Hydrogen peroxide, diacetyl, acetoin, and bacteriocins	Antimicrobial activity	Damodharan, Palaniyandi, Yang, and Suh (2015).
Sauerkraut	*Lactococcus lactis*	Bacteriocin-nisin,	Anti-bacterial, anti-inflammatory, anti-carcinogenic, anti-oxidative, gut microbial and immuno- modulatory.	Mir et al., 2018, Peňas et al., 2017, Ai et al., 2016, Noh et al., 2016, Peñas et al., 2012.
	*Leuconostoc mesenteroides*	Ascorbigen, Sulforaphane and Glucosinolates (isothiocyanates, nitriles, epithionitriles and thiocyanates)	Anti-carcinogenic properties; good source of Vitamin C and E.	Park et al., 2017, Nugrahedi et al., 2015, Peñas et al., 2010.
		Indol-3-carbinol and Allyl isothiocyanate	Anti-inflammatory properties	Wagner, Boesch-Saadatmandi, Dose, Schultheiss, and Rimbach (2012).
		Phenyl isothiocyanate and allyl isothiocyanate	Anti-oxidant properties	Manesh and Kuttan (2003).
		D-phenyllactic acid	Anti-bacterial properties	Peters et al. (2019).
	*Lactobacillus paracasei* HD1-7	Paracin 1.7	Anti-microbial properties	Ge, Sun, Xin, Wang, and Ping (2016).
Fermented cucumber (cucumber pickles)	*Lactiplantibacillus plantarum* C19	Plantaricin C19	Anti-bacterial activity against *Listeria grayi*	Mir et al. (2018).
	*Pediococcus pentosaceus* CRAG3	Dextran	Anti-cancer properties	Shukla and Goyal (2013).
	*Lactobacillus, Pediococcus and Leuconostoc isolates*	Plantaricin A, pediocin, enterocin, nisin and mesentericin,A	Anti-bacterial effect against pathogenic *Listeria* and Gram-positive bacteria	Singh and Ramesh (2008).
		Leucine-proline-proline (0.30–0.33 mg/kg), isoleucine-proline-proline (0.42–0.49 mg/kg), valine-proline-proline (0.32–0.35 mg/kg) andLysine-proline (0.93–1.5 mg/kg)	Angiotensin-Converting Enzyme (ACE) inhibitory activities	Fideler, Johanningsmeier, Ekelöf, and Muddiman (2019).
		Gamma-aminobutyric acid (GABA)	Anti-hypertensiveImmune supportAnti-anxiety	Moore, DuVivier, and Johanningsmeier (2021).
Fermented olives	*Lactobacillus pentosus* B96	Bacteriocins	Anti-bacterial activity against *Weissella mesenteroides*	Delgrado *et al*. (2005).
	*Lactobacillus plantarum* NC8	Bacteriocins	Anti-bacterial activities against*Helicobacter pylori*, *Propionibacterium* spp. and *Clostridium perfringens*	Ruiz-Barba, Caballero-Guerrero, Maldonado-Barragán, and Jiménez-Díaz (2010).
	*Lactobacillus plantarum*	Tyrosol and hydroxytyrosol	Anti-microbial and anti-oxidant properties	Benincasa, Muccilli, Amenta, Perri, and Romeo (2015).
Fermented carrot	*Lactobacillus* strains	Bacteriocins	Anti-bacterial activities against *Bacillus cereus, Staphylococcus aureus* and *E. coli*	Joshi, Sharma, and Rana (2006).
	*Lactobacillus rhamnosus* GG (LGG)	Free phenolics	Anti-oxidants	Hu et al. (2019).
	*Lactobacillus plantarum*	Short-chain fatty acid (SCFA)	Anti-diabetic	Wan et al., 2019, Li et al., 2014.
Kimchi	*Lactobacillus sakei*	Bacteriocin,Sakacin C2, benzyl isothiocyanate, indole compounds, thiocyanate and b-sitosterol.	Anti-bacterial activities against *Staphylococcus aureus* ATCC 63,589 and *E. coli.*Anti-obesogenic, anti-cancerous, anti-inflammatory, anti-oxidant, anti-hypertensive, anti-ageing, anti-constipation, gut microbial and immuno- modulatory.	Peters et al., 2019, Peňas et al., 2017, Ai et al., 2016, Noh et al., 2016, Peñas et al., 2012, Gao et al., 2010.
	*Leuconostoc mesenteroides* LBP-K06	Cyclo (Ser-Pro), cyclo (Tyr-Pro), and cyclo (Leu-Pro)	Anti-microbial activity	Liu, Kim, Kwak, and Kang (2017).
	*Bacillus amyloliquefaciens* CBSYD1	YD1(peptide rich in glycin)	Anti-microbial activity against Gram-positive, Gram-negative, resistant bacteria, and Anti-oxidant activities	Rahman et al. (2017).
	*Leuconostoc citreum* GJ7 and *Lactococcus lactis* BH5	Bacteriocins	Anti-microbial activity	Rahman et al. (2017).
	*Pediococcus pentosaceus*	Pediocins	Anti-microbial activity	Shin, Han, Ryu, Kim, and Lee (2008);
	*Lactococcus lactis* subsp. Lactis A164	Nisin-like bacteriocin	Anti-microbial activity against *Staphylococcus aureus*, *Listeria monocytogenes* and *Salmonella typhimurium*	Choi, Cheigh, Kim, and Pyun (2000).
		β-sitosterol, thiocyanate and benzyl isothiocyanate	Anti-oxidative, anti-carcinogenic, anti-inflammatory, anti-ageing, anti-atherosclerotic, anti-obesity, anti-constipation, anti-hypertensive and anti-diabetic and lipid-lowering activities	Park et al. (2017).
		KIMCHI3-(40-Hydroxyl-30,50-dimethoxyphenyl) propionic acid	Anti-inflammatory effect	Jeong et al. (2015).
		Dichloromethane, chlorophyll,carotenoids, phenolics and vitamin C, capsaicin, quercetin, and 3-(4′-hydroxyl-3′,5′-dimethoxyphenyl) propionic acid	Anti-oxidative activity	Woo et al., 2017, Hwang and Song, 2001.
	*Leuconostoc citreum*	Compound K	Tumour suppressor	Quan et al. (2011);
Inziangsang	*Lactiplantibacillus plantarum* IB2	Bacteriocin	Anti-bacterial activity against *Staphylococcus aureus* S1	Tamang, Tamang, Schillinger, Guigas, and Holzapfel (2009).
Gundruk	*Lactobacillus spicheri* G2	Bacteriocin	Anti-microbial activity against *Streptococcus mutans*, *Staphlococcus aureus*, *Listeria monocytogenes*, *Clostridium perfringens*, *Lactobacillus plantarum*, *Bacillus cereus* and *Leuconostoc mesenteroides.* A good appetizer, source of Vitamins B and C, lactic acids, carotene, amino acids and minerals, anti-cancerous	Karki et al., 2016, Gautam and Sharma, 2015, Tamang et al., 2016, Tamang and Tamang, 2010, Tamang et al., 2009.
Suan-Tsai	*Lactiplantibacillus plantarum* JLA-9	Plantaricin	Anti-bacterial activity against *Bacillus* spp.	Zhao et al. (2016).
Nozawana-zuke	*Lactobacillus curvatus* and *Lactiplantibacillus plantarum*	Interferon-gamma (IFN-γ) andInterleukin 10 (IL-10).	Immunomodulatory activities	Sandagdorj, Hamajima, Kawahara, Watanabe, and Tanaka (2019).
Soidon	*Bacillus subtilis, Bacillus cereus, Bacillus pumilus, Lactobacillus brevis, Lactobacillus plantarum, Carnobacterium* sp*., Enterococcus faecium,* and *Pseudomonas fluorescens*	Amino acids	Anti-oxidative, anti-cancer, anti-microbial, anti-ageing, and immunoregulatory, anti-obesogenic, a good source of vitamins C and E	Thakur et al., 2016, Jeyaram et al., 2010.
Sinki	*Lactobacillus plantarum, Lactobacillus brevis,* and *Lactobacillus fermentum*	Amino acids	Indigestion remedy, a good appetizer, cures stomach pains and diarrhoea	Das et al., 2016, Karki et al., 2016.
Inziangsang	*Lactobacillus plantarum*	_	Anti-bacterial (*Pseudomonas aeruginosa* and *Staphylococcus aureus*), a good appetizer, and aids digestion	Tamang and Tamang (2009).
Khalpi	*Lactobacillus plantarum, Lactobacillus brevis* and *Leuconostoc fallax*	Bacteriocin and amino acids	Improved palatability, a good appetizer, detoxification of virulent/toxic synthesis and degeneration of mycotoxins	Behera, El Sheikha, Hammami, and Kumar (2020).
Kanji	*Lactobacillus plantarum, Lactobacillus delbrueckii, Lactobacillus curvatus* and *Lactobacillus coryniformis*	_	Hepatoprotective, diuretic properties, uterine-stimulating, anti-tumour, improves appetite, digestion, anti-infection, has cooling and soothing properties	Karki et al., 2016, Kingston et al., 2010, HALLIWELL, 2007, Sura et al., 2001.

### 3.2 Proteomic analysis of vegetables

Vegetables, just like fish products, are also considered a health-beneficial diet type; they have high nutrient and water activity content. These factors make them easily liable to microbial and enzymatic degradations (Sajjad, Rasool, Ahmad Fazili, & Ahmed Bhat, 2000), hence their short shelf life. Numerous works on plant-sourced foods show that they contain various bioactive substrates, either nutritive or anti-nutritive (Sharma et al., 2020, Awak et al., 2017). Notwithstanding, the microorganisms involved in plant-food fermentation increase the nutritional contents of fermented plant products by (a) increasing the amount and bioavailability of nutrients and (b) enhancing the density of nutrients (Nkhata, Ayua, Kamau, & Shingiro, 2018). They enhance the density of nutrients by synthesizing promoters that aid with nutrient adsorption, influence the uptake of nutrients via the mucosa membrane, pre-digest the food components, and reduce or degrade the anti-nutrient contents of the parent food. Bacteria from the *Lactococcus* family are the most identifiable microbes associated with plant fermentation.

According to Voidarou et al. (2021), fermentation, an age-long process, is gaining keen interest. Many beneficial end-product possibilities are obtainable as long as microorganisms and food substrates are placed side by side in conducive environments (anaerobic). The end-products from vegetable-carbohydrate fermentation are as varied as many types of vegetables and varieties of fermenting microorganisms (Table 1). Plant carbohydrate fermentation entails accepting electrons by organic molecules (pyruvate or acetyl CoA) and reactions that lead to the formation of important peptides. Peptides from fermented vegetables can be used as microbial energy sources; howbeit, they are not the preferred energy molecule. Instead, they are used for the production of hormones, enzymes, and haemoglobin; needed for cell growth and repairs, the normal functioning of the muscles, nerve signaling, and immunity (Marsh, Munn, & Baines, 2013), making plant-sourced peptides an important set of macromolecules for body functions. Fermented vegetable proteins are linked with decreased risk for metabolic diseases (Marsh et al., 2013); this is due to plant-sourced diets having very low saturated fats, cholesterol-free, good sources of antioxidants, high fiber contents, and haem iron. Table 1 enlists works with immunomodulatory benefits of peptides from fermented vegetables.

## 4 Proteomics of microbes associated with fermented foods

Metaproteomic studies of any fermented food give insights into microbial type, community interactions, typical roles, and expressed protein molecules (Ji et al., 2017). The demand for fermented products with high consistency by urban dwellers is turning food industries into controlled fermentation processes; however, rural dwellers still prefer traditional methods (Tamang et al., 2020, Tamang, Shin, Jung, & Chae*,* 2016). About 90% of consumed fermented products are prepared uncontrolled in homes utilizing inherent food microbes. The mixture is left on its own or given conducive environmental conditions to thrive. Traditionally fermented foods are generally simple, require simple ingredients, minimal preparations, and processing (Marco et al., 2017); however, the diversity of fermenting microbial succession involved is very complex, notwithstanding.

In every natural microbe-food substrate mixture, there is also a mixture of functional and non-functional microorganisms. The functional microbes are responsible for the biotransformation of the chemical constituents in food substrates (Tamang, Shin, Jung, & Chae, 2016). The fermentation process, whether traditional or industrial, follow three distinguished units of operations thus: (a) pre-treatment of the food substrates, which includes transportation, salting, grading, washing, sorting, mixing, etc. (b) the bioprocessing that utilizes suitable microbes/enzymes that bio-transform, synthesize, remove, degrade, etc., certain substrates and (c) bioprocessing atmosphere, that includes cooling, freezing, and heating.

Stability in the composition of microbes is an important factor, as alterations in their diversity during the process might yield noticeable differences in organoleptic properties/quality- even with the same food substrate. Therefore, to have consistent, high-quality, safe, and good sensory fermented end-product(s), the microbial composition must be stable and resilient (Marco et al., 2017). Modern fermentation has progressed towards end-product consistencies, with certain process modifications. Portions of previously fermented foods (like fruits or malted cereals with fermenting microbes) can be added to raw food substrates or new food batches to initiate fermentation. This method termed back slopping is frequently used to standardized microbial fermented end-products (Tamang et al., 2020). Standardized starter cultures allow for consistent fermented end-products, reduced spoilage, increased food safety, and ensures large-scale production of fermentation end-products (Tamang et al., 2020). Just as with those functional microorganisms in traditional/natural fermentation, starter microbial cultures transform the raw foods and yield more desirable/healthier end-products.

Fermented foods (fish and vegetables inclusive) are studied using different methods. The principal is the conventional microbiological culture-dependent methods, where isolates are identified using phenotypic and biochemical characterizations. However, microbes are now identified using molecular methods like metagenomics and massive sequencing. Lactic acid bacteria (LABs), such as *Leuconostoc*, *Lactobacillus*, and *Weissella* species (Chen, Chen, and Lei, 2017), are the prominent bacterial species that help ferment vegetables. According to Lee, Jung, & Jeon (2015), genomics and other technologies have been used to study the dynamic microbial communities and metabolic changes during fermentations. The functions of the peptides make fermentation end-products a vital patterned food type. Yang, Fan, and Xu (2020) identified about 2,175 proteins in fermented *Siniperca chuatsi*, 1,217 of which were involved in metabolic pathways, while 352 were associated with amino acid metabolism. Certain microbes such as *Streptococcus* sp., *Bacillus* sp., *Escherichia* sp., and *Pseudoalteromonas* sp. possess about 63 amino acids that are degradation-related, all of which generate aromatic compounds. These compounds are responsible for the unique taste, flavour, and organoleptic attributes of fermented foods (Yang et al., 2020).

## 5 Challenges and future perspectives

Some challenges hamper the application of metaproteomic analysis on traditionally fermented foods (fish and vegetables); these include but are not limited to the redundancy of protein identifications, impurities, and complexities of food samples, paucity of genome sequences essential for their protein identifications. Additionally, uneven distribution of species, extensive fluctuations in expression levels of proteins in microorganisms, and significant genetic varieties within microbial communities are other major challenges in metaproteomic studies (Simon & Daniel, 2011). Furthermore, identifying proteins relying on a metagenomics database derived from the same sample and any sequence cross-contamination or submission errors onto the metagenomics database may compromise homologous protein identification (Pible & Armengaud, 2015). Therefore, care must be exercised throughout the entire process.

Other concerns are adequate and comprehensive protein separation, *meta*-information from online sources, and redundant protein groupings. These also need attention as they can affect the outcomes. Advanced methods need to be employed for the extraction, identification, and verification methods of metaproteomic studies to be effective. Although omics technologies have offered unique datasets at a variety of molecular levels (Lagier et al., 2018), metaproteomics and other omics approaches, on the other hand, cannot fully reveal the presence and growth of a microbial community. Due to the vast extreme complexities of samples from traditionally fermented fish and vegetables, systematic biology can provide a whole picture of activities and higher-level biological relationships by merging multi-omics data and bioinformatics technology to link cause and effect. Consequently, the molecular nature of biological activities is revealed (Cocolin et al., 2018), thereby obtaining new functional microorganisms as well as functional metabolites in fermented fish and vegetables. Furthermore, for effectiveness, metaproteomics is not a stand-alone technique, it should be combined with cytometry, microscopy, *meta*-transcriptomics, metabolomics, and metagenomics for thorough investigations and understanding of microbial populations and metabolic models (Vilanova & Porcar, 2016).

Protein modification and interaction should be another point of interest, especially with traditional fermentation (Gagnaire, Jardin, Jan, and Lortal, 2009). Additional researches into regulatory links between proteins’ post-translational modifications and metabolites, as well as proteins interactions, can help to elucidate the regulatory mechanisms of proteins and product quality in traditionally fermented foods. These interactions and modifications are capable of altering fermentation outcomes and processes.

The development of advanced software tools with features to handle enormous datasets and are user-friendly can greatly improve metaproteomic analyses. The provision of cheaper metaproteomic analytic tools, just like DNA sequencing, would make analysis commonplace (Chiapello, Zampieri, & Mello, 2020) and enhance the listing of more novel proteins never before reported/explored in traditional or controlled fermentations.

## 6 Conclusion

Fermentation is an important process, not just because it preserves food. However, it further produces various molecules and metabolites that make the end-products healthier than the initial raw food substrates, all thanks to versatile microorganisms and conducive environments. Some of the end-product proteins are signature organoleptic, preservative, and anti-microbial peptides. Therefore, increased detailed data from studies are needed to elucidate the microecology responsible for the production of peptides, their accurate identification, characteristics, and functions in fermented fish and vegetables (Tamang et al., 2020). The elucidation would enable a more controlled production of these important peptides on a large scale.

Fermentation is a process that will always be with us. With the arrays of microbes identified in fermented fish and vegetable products (although studies on fermented fish peptides are few), researchers can enlist important molecules associated with anti-microbial, anti-obesity, anti-oxidative, anti-hypertensive, even protein molecules that inhibit the activities of ACE- a host receptor that viral agents attach for host entry and replication. Nutraceutical firms can harvest these proteins and formulate them into good foods. Food and pharmaceutical scientists can solve the need for natural therapeutics to abate certain diseases via fermentation and harvesting these sets of peptides from healthy raw foods (fish and vegetables).

## Funding

This research did not receive any specific grant from funding agencies in the public, commercial, or not-for-profit sectors.

## CRediT authorship contribution statement

**Emmanuel Sunday Okeke:** Data curation, Resources, writing-original draft. Richard Ekeng Ita: Data curation, Resources, writing-original draft. **Egong John Egong:** Data curation, Resources, writing-original draft. **Lydia Etuk Udofia:** Data curation, Resources, writing-original draft. **Chiamaka Linda Mgbechidinm:** Conceptualization, Writing-review and editing, and visualization, Data curation, Resources, writing-original draft. **Otobong Donald Akan:** Data curation, Resources, writing-original draft, Project Administration, Supervision, and Writing-review and editing.

## Declaration of Competing Interest

The authors declare that they have no known competing financial interests or personal relationships that could have appeared to influence the work reported in this paper.

## References

[b0005] Ai C., Ma N.a., Zhang Q., Wang G., Liu X., Tian F., Yodoi J. (2016). Immunomodulatory effects of different lactic acid bacteria on allergic response and its relationship with *in vitro* properties. PLoS ONE.

[b0010] Anguraj Vadivel A.K. (2015). Gel-based proteomics in plants: Time to move on from the tradition. Frontiers in plant science.

[b0015] Arya M., Prakash S., Sougrakpam Y., Deswal R. (2021). *Brassica juncea* leaf cuticle proteome analysis shows myrosinase protein, antifreeze activity, and post-translationally modified secretory proteins. Plant Physiology and Biochemistry.

[b0020] Awak E.E., Udofia O.E., Akan O.D., Uffia I., Udoekong N.S. (2017). Proximate and Anti-nutrient Compositions of Cocoyam (*Colocasia esculenta*): The Effect of Cooking and Dietary Palm Oil Treatments. International Journal of Biochemistry Research & Review.

[b0025] Behera S.S., El Sheikha A.F., Hammami R., Kumar A. (2020). Traditionally fermented pickles: How the microbial diversity associated with their nutritional and health benefits?. Journal of Functional Foods.

[b0030] Benincasa C., Muccilli S., Amenta M., Perri E., Romeo F.V. (2015). Phenolic trend and hygienic quality of green table olives fermented with *Lactobacillus plantarum* starter culture. Food Chemistry.

[b0035] Calderón-González K.G., Hernández-Monge J., Herrera-Aguirre M.E., Luna-Arias J.P. (2016). Bioinformatics Tools for Proteomics Data Interpretation. Advances in Experimental Medicine and Biology.

[b0040] Chaudhary A., Bhalla S., Patiyal S., Raghava G.P.S., Sahni G. (2021). FermFooDb: A database of bioactive peptides derived from fermented foods. Heliyon.

[b0045] Chen G., Chen C., Lei Z. (2017). Meta-omics insights in the microbial community profiling and functional characterization of fermented foods. Trends in Food Science & Technology.

[b0050] Chen C., Hou J., Tanner J.J., Cheng J. (2020). Bioinformatics Methods for Mass Spectrometry-Based Proteomics Data Analysis. International Journal of Molecular Sciences.

[b0055] Chen J., Wang F., Liu Z., Liu J., Zhu Y., Zhang Y., Zou H. (2017). Electrospray ionization in concentrated acetonitrile vapor improves the performance of mass spectrometry for proteomic analyses. Journal of Chromatography A.

[b0060] Chiapello M., Zampieri E., Mello A. (2020). A small effort for researchers, a big gain for soil metaproteomics. Frontiers in Microbiology.

[b0070] Choi H.J., Cheigh C.I., Kim S.B., Pyun Y.R. (2000). Production of a nisin-like bacteriocin by *Lactococcus lactis* subsp. Lactis A164 isolated from Kimchi isolated from Kimchi. Journal of Applied Microbiology.

[b0075] Cocolin L., Mataragas M., Bourdichon F., Doulgeraki A., Pilet M.-F., Jagadeesan B., Phister T. (2018). Next-generation microbiological risk assessment meta-omics: The next need for integration. International Journal of Food Microbiology.

[b0080] Colignon B., Raes M., Dieu M., Delaive E., Mauro S. (2013). Evaluation of three-dimensional gel electrophoresis to improve quantitative profiling of complex proteomes. Proteomics.

[b0085] Damodharan K., Palaniyandi S.A., Yang S.H., Suh J.-W. (2015). *In vitro* probiotic characterization of *Lactobacillus* strains from fermented radish and their anti-adherence activity against enteric pathogens. Canadian Journal of Microbiology.

[b0090] Das G., Patra J.K., Singdevsachan S.K., Gouda S., Shin H. (2016). Diversity of traditional and fermented foods of the Seven Sister states of India and their nutritional and nutraceutical potential: A review. Frontiers in Life Science.

[b0100] FAO, SOFIA, (2014). The state of world fisheries and aquaculture. *In*: Opportunities and Challenges. ISBN: 978-92-5-108275-1.

[b0105] Fideler J., Johanningsmeier S.D., Ekelöf M., Muddiman D.C. (2019). Discovery and quantification of bioactive peptides in fermented cucumber by direct analysis IR-MALDESI mass spectrometry and LC-QQQ-MS. Food Chemistry.

[b0110] Gagnaire V., Jardin J., Jan G., Lortal S. (2009). Invited review: Proteomics of milk and bacteria used in fermented dairy products: From qualitative to quantitative advances. Journal of Dairy Science.

[b0115] Galindo-Luján Rocío, Pont Laura, Minic Zoran, Berezovski Maxim V., Sanz-Nebot Victoria, Benavente Fernando (2021). Characterization and differentiation of quinoa seed proteomes by label-free mass spectrometry-based shotgun proteomics. Food Chemistry.

[b0120] Gao Y., Jia S., Gao Q., Tan Z. (2010). A novel bacteriocin with a broad inhibitory spectrum produced by *Lactobacillus sake* C2, isolated from traditional Chinese fermented cabbage. Food Control.

[b0125] Gautam Neha, Sharma Nivedita (2015). A study on characterization of new bacteriocin produced from a novel strain of *Lactobacillus spicheri* G2 isolated from Gundruk- a fermented vegetable product of North East India. Journal of Food Science and Technology.

[b0130] Ge J., Sun Y., Xin X., Wang Y., Ping W. (2016). Purification and Partial Characterization of a Novel Bacteriocin Synthesized by *Lactobacillus paracasei* HD1-7 Isolated from Chinese Sauerkraut Juice. Scientific Reports.

[b0135] Geron Augustin, Werner Johannes, Wattiez Ruddy, Lebaron Philippe, Matallana-Surget Sabine (2019). Deciphering the functioning of microbial communities: Shedding light on the critical steps in metaproteomics. Frontiers in Microbiology.

[b0140] Halliwell B (2007). Dietary polyphenols: Good, bad, or indifferent for your health?. Cardiovascular Research.

[b0145] Hart-Smith G., Reis R.S., Waterhouse P.M., Wilkins M.R. (2017). Improved Quantitative Plant Proteomics via the Combination of Targeted and Untargeted Data Acquisition. Frontiers in Plant Science.

[b0150] Hayoun K., Gouveia D., Grenga L., Pible O., Armengaud J., Alpha-Bazin B. (2019). Evaluation of Sample Preparation Methods for Fast Proteotyping of Microorganisms by Tandem Mass Spectrometry. Frontiers in Microbiology.

[b0155] Heyer Robert, Schallert Kay, Büdel Anja, Zoun Roman, Dorl Sebastian, Behne Alexander, Benndorf Dirk (2019). A Robust and Universal Metaproteomics Workflow for Research Studies and Routine Diagnostics Within 24 h Using Phenol Extraction, FASP Digest, and the MetaProteomeAnalyzer. Frontiers in Microbiology.

[b0160] Heyer R., Schallert K., Zoun R., Becher B., Saake G., Benndorf D. (2017). Challenges and perspectives of metaproteomic data analysis. Journal of Biotechnology.

[b0165] Hu Rongkang, Zeng Feng, Wu Linxiu, Wan Xuzhi, Chen Yongfang, Zhang Jiachao, Liu Bin (2019). Fermented carrot juice attenuates type 2 diabetes by mediating gut microbiota in rats. Food and Function.

[b0170] Hwang J.W., Song Y.O. (2001). The effects of solvent fractions of kimchi on plasma lipid concentration of rabbit-fed high-cholesterol diet. Journal of the Korean Society of Food Science and Nutrition.

[b0175] Pilot system recovers protein, lipids from fish byproducts. *Global Aquaculture Advocate* (https://www.aquaculturealliance.org/advocate/pilot-system-recovers-protein-lipids-from-fish-byproducts/).

[b0180] Jeong J.W., Choi I.W., Jo G.H., Kim G.Y., Kim J., Suh, H.,….Choi, Y. H. (2015). Anti-inflammatory effects of 3-(4 *0*-Hydroxyl-3 *0*, 5 *0*-dimethoxyphenyl) propionic acid, an active component of Korean cabbage kimchi, in lipopolysaccharide-stimulated bv2 microglia. Journal of Medical Food.

[b0185] Jeyaram K., Romi W., Singh T.A., Devi A.R., Devi S.S. (2010). Bacterial species associated with traditional starter cultures used for fermented bamboo shoot production in Manipur state of India. International Journal of Food Microbiology.

[b0190] Ji Chaofan, Zhang Jingbo, Lin Xinping, Han Jing, Dong Xiuping, Yang Song, Zhu Beiwei (2017). Metaproteomic analysis of microbiota in the fermented fish, *Siniperca chuatsi*. LWT-Food Science and Technology.

[b0195] Joshi V.K., Sharma S., Rana N.S. (2006). Production, purification, stability and efficacy of bacteriocin from isolates of natural lactic acid fermentation of vegetables. Food Technology and Biotechnology.

[b0200] Kailasa Suresh Kumar, Koduru Janardhan Reddy, Baek Seung Hoon, Wu Hui-Fen, Hussain Chaudhery Mustansar, Park Tae Jung (2020). Review on matrix-assisted laser desorption/ionization time-of-flight mass spectrometry for the rapid screening of microbial species: A promising bioanalytical tool. Microchemical Journal.

[b0205] Karki T., Ojha P., Panta O.P., Tamang J.P. (2016). Ethnic Fermented Foods and Alcoholic Beverages of Asia.

[b0220] Kingston J.J., Radhika M., Roshini P.T., Raksha M.A., Murali H.S., Batra H.V. (2010). Molecular characterization of lactic acid bacteria recovered from natural fermentation of beetroot and carrot Kanji. Indian Journal of Microbiology.

[b0225] Krutz, N. L.,Winget, J., Ryan, C. A.,Wimalasena, R., Maurer-Stroh, S., Dearman, R. J., …. Gerberick, G. F. (2019). Proteomic and bioinformatic analyses for the identification of proteins with low allergenic potential for hazard assessment. *Toxicological Sciences*, 170(1), 210–222. https://doi.org/10.1093/toxsci/kfz078. 10.1093/toxsci/kfz078 30903174

[b0230] Kunath B.J., Minniti G., Skaugen M., Hagen L.H., Vaaje-Kolstad G., Eijsink V.G., Arntzen M.O., Capelo-Martinez J.-.L. (2019). Emerging Sample Treatments in Proteomics.

[b0235] Lagier J.C., Dubourg G., Million M., Cadoret F., Bilen M., Fenollar F., Raoult D. (2018). Culturing the human microbiota and culturomics. Nature Reviews Microbiology.

[b0240] Lee S.H., Jung J.Y., Jeon C.O. (2015). Source Tracking and Succession of Kimchi Lactic Acid Bacteria during Fermentation. Journal of Food Science.

[b0245] Li C., Ding Q., Nie S., Zhang Y., Xiong T., Xie M. (2014). Carrot juice fermented with *Lactobacillus plantarum* NCU116 ameliorates Type 2 Diabetes in rats. Journal of Agriculture and Food Chemistry.

[b0265] Liu N., Qin L., Mazhar M., Miao S. (2021). Integrative transcriptomic-proteomic analysis revealed the flavor formation mechanism and antioxidant activity in rice-acid inoculated with *Lactobacillus paracasei* and *Kluyveromyces marxianus*. Journal of Proteomics.

[b0250] Liu R., Kim A.H., Kwak M., Kang S. (2017). Proline-Based Cyclic Dipeptides from Korean Fermented Vegetable Kimchi and from *Leuconostoc mesenteroides* LBP-K06 Have Activities against Multidrug-Resistant Bacteria. Frontiers in Microbiology.

[b0255] Liu S., Li Z., Yu B., Wang S., Shen Y., Cong H. (2020). Recent advances on protein separation and purification methods. Advances in Colloid and Interface Science.

[b0260] Liu J., Lin C., Zhang W., Yang Q., Meng J., He L., Zeng X. (2021). Exploring the bacterial community for starters in traditional high-salt fermented Chinese fish (Suanyu). Food Chemistry.

[b0270] Manesh C., Kuttan G. (2003). Anti-tumour and anti-oxidant activity of naturally occurring isothiocyanates. Journal of Experimental and Clinical Cancer Research.

[b0275] Marco Maria L, Heeney Dustin, Binda Sylvie, Cifelli Christopher J, Cotter Paul D, Foligné Benoit, Hutkins Robert (2017). Health benefits of fermented foods: Microbiota and beyond. Current Opinion in Biotechnology.

[b0280] Marsh K.A., Munn E.A., Baines S.K. (2013). Protein and vegetarian diets. Medical Journal of Australia.

[b0285] Méndez L., Pazos M. (2017). In: Proteomics in Food Science.

[b0290] Mir S.A., Raja J., Masoodi F.A. (2018). Fermented vegetables, a rich repository of beneficial probiotics-a review. Fermentation Technology.

[b0295] Moore Jennifer Fideler, DuVivier Rachel, Johanningsmeier Suzanne D. (2021). Formation of γ-aminobutyric acid (GABA) during the natural lactic acid fermentation of cucumber. Journal of Food Composition and Analysis.

[b0300] Muth T., Renard B.Y., Martens L. (2016). Metaproteomic data analysis at a glance: Advances in computational microbial community proteomics. Expert Review of Proteomics.

[b0305] Nkhata S.G., Ayua E., Kamau E.H., Shingiro J.B. (2018). Fermentation and germination improve nutritional value of cereals and legumes through activation of endogenous enzymes. Food science & nutrition.

[b0310] Noh, B. S., Seo, H. Y., Park, W. S., & Oh, S. (2016). Chapter 19—Safety of Kimchi. In: *Regulating Safety of Traditional and Ethnic Foods*; Prakash, V., Mart ín-Belloso, O., Keener, L., Astley, S., Braun, S., McMahon, H., Lelieveld, H. (eds.). San Diego, CA, USA: Academic Press, pp. 369-380. https://doi.org/10.1016/B978-0-12-800605-4.00019-0.

[b0315] Nugrahedi Probo Y., Verkerk Ruud, Widianarko Budi, Dekker Matthijs (2015). A mechanistic perspective on process-induced changes in glucosinolate content in *Brassica* vegetables: A review. Critical Reviews in Food Science and Nutrition.

[b0320] Olovo, C. V., Udoekong, N. S., & Akan. O. D. (2021). Precision Nutrition, Diet and Gut-Microbiota in Obesity. Journal of Biotechnology and Bioresources, 2, 1-3. https://crimsonpublishers.com/jbb/pdf/JBB.000549.pdf.

[b0325] Ortea I., O’Connor G., Maquet A. (2016). Review on proteomics for food authentication. Journal of Proteomics.

[b0330] Park, K. Y., Kim, H. Y., & Jeong, J. K. (2017). Chapter 20—Kimchi and Its Health Benefits. *In*: *Fermented Foods in Health and Disease Prevention*; Frias, J., Martinez-Villaluenga, C., Peñas, E., (eds.). Boston, MA, USA: Academic Press, pp. 477-502. https://doi.org/10.1016/B978-0-12-802309-9.00020-0.

[b0340] Peñas Elena, Frias Juana, Sidro Beatriz, Vidal-Valverde Concepción (2010). Chemical evaluation and sensory quality of sauerkrauts obtained by natural and induced fermentations at different NaCl levels from *Brassica oleracea* Var. Capitata Cv. Bronco grown in eastern Spain. Effect of storage. Journal of Agricultural and Food Chemistry.

[b0345] Peňas, E., Martinez-Villaluenga, C., & Frias, J. (2017). Sauerkraut: Production, composition, and health benefits. *In: Fermented Foods in Health and Disease Prevention*. Martinez-Villaluenga, C., Peñas, E. (eds.) Academic Press: Boston, MA, USA pp. 557-576. https://doi.org/10.1016/B978-0-12-802309-9.00024-8.

[b0350] Peñas Elena, Martinez-Villaluenga Cristina, Frias Juana, Sánchez-Martínez Maria José, Pérez-Corona Maria Teresa, Madrid Yolanda, Vidal-Valverde Concepción (2012). Se improves indole glucosinolate hydrolysis products content, Se-methylselenocysteine content, antioxidant capacity and potential anti-inflammatory properties of sauerkraut. Food Chemistry.

[b0355] Peters Anna, Krumbholz Petra, Jäger Elisabeth, Heintz-Buschart Anna, Çakir Mehmet Volkan, Rothemund Sven, King Nicole (2019). Metabolites of lactic acid bacteria present in fermented foods are highly potent agonists of human hydroxycarboxylic acid receptor 3. PLoS Genetics.

[b0360] Pible O., Armengaud J. (2015). Improving the quality of genome, protein sequence, and taxonomy databases: A prerequisite for microbiome meta-omics 2.0. Proteomics.

[b0365] Quan Q., Feng J., Lui L., Shi T., Chu I.K. (2017). Phosphoproteome of crab-eating macaque cerebral cortex characterized through multidimensional reversed-phase liquid chromatography/mass spectrometry with tandem anion/cation exchange columns. Journal of Chromatography A.

[b0370] Quan L., Piao J., Min J., Yang D., Lee H.N., Yang D.C. (2011). Bioconversion of ginsenoside Rb1 into compound K by *Leuconostoc citreum* LH1 isolated from kimchi. Brazilian Journal of Microbiology.

[b0375] Rahman M.D., Choi Y.H., Choi Y.S., Alam M.B., Lee S.H., Yoo J.C. (2017). A novel antioxidant peptide, purified from *Bacillus amyloliquefaciens*, showed strong antioxidant potential via Nrf-2 mediated heme oxygenase-1 expression. Food Chemistry.

[b0380] Ruiz-Barba José Luis, Caballero-Guerrero Belén, Maldonado-Barragán Antonio, Jiménez-Díaz Rufino (2010). Coculture with specific bacteria enhances survival of *Lactobacillus plantarum* NC8, an autoinducer-regulated bacteriocin producer, in olive fermentations. Food Microbiology.

[b0385] Sajjad N., Rasool A., Ahmad Fazili A.B., Ahmed Bhat E. (2000). Fermentation of Fruits and Vegetables. Plant Archives.

[b0390] Sandagdorj B., Hamajima C., Kawahara T., Watanabe J., Tanaka S. (2019). Characterization of Microbiota that Influence Immunomodulatory Effects of Fermented *Brassica rapa* L. Microbes and Environments.

[b0395] Sharma R., Garg P., Kumar P., Bhatia S.K., Kulshrestha S. (2020). Microbial Fermentation and Its Role in Quality Improvement of Fermented Foods. Fermentation.

[b0400] Shin M.S., Han S.K., Ryu J.S., Kim K.S., Lee W.K. (2008). Isolation and partial characterization of a bacteriocin produced by *Pediococcus pentosaceus* K23–2 isolated from kimchi. Journal of Applied Microbiology.

[b0405] Shukla R., Goyal A. (2013). Novel dextran from *Pediococcus pentosaceus* CRAG3 isolated from fermented cucumber with anti-cancer properties. International Journal of Biological Macromolecules.

[b0410] Sim Kae Hwan, Liu Lillian Chia-Yi, Tan Hwee Tong, Tan Kelly, Ng Daniel, Zhang Wei, Bi Xuezhi (2020). A comprehensive CHO SWATH-MS spectral library for robust quantitative profiling of 10,000 proteins. Scientific Data.

[b0415] Simon C., Daniel R. (2011). Metagenomic analyses: Past and future trends. Applied and Environmental Microbiology.

[b0420] Singh Atul Kumar, Ramesh Aiyagari (2008). Succession of dominant and antagonistic lactic acid bacteria in fermented cucumber: Insights from a PCR-based approach. Food Microbiology.

[b0425] Song Ehwang, Gao Yuqian, Wu Chaochao, Shi Tujin, Nie Song, Fillmore Thomas L., Liu Tao (2017). Targeted proteomic assays for quantitation of proteins identified by proteogenomic analysis of ovarian cancer. Scientific Data.

[b0430] Sura K., Garg S., Garg F.C. (2001). Microbiological and biochemical changes during fermentation of Kanji. Journal of Food Science and Technology.

[b0435] Takemori N., Takemori A., Ishizaki J., Hasegawa H. (2014). Enzymatic protein digestion using a dissolvable polyacrylamide gel and its application to mass spectrometry-based proteomics. Journal of Chromatography B: Analytical Technologies in the Biomedical and Life Sciences.

[b0440] Tamang Jyoti Prakash, Cotter Paul D., Endo Akihito, Han Nam Soo, Kort Remco, Liu Shao Quan, Hutkins Robert (2020). Fermented foods in a global age: East meets West. Comprehensive Reviews in Food Science and Food Safety.

[b0445] Tamang B., Tamang J.P. (2009). Traditional knowledge of biopreservation of perishable vegetables and bamboo shoots in Northeast India as food resources. Indian Journal of Traditional Knowledge.

[b0450] Tamang B., Tamang J.P. (2010). *In situ* fermentation dynamics during production of gundruk and khalpi, ethnic fermented vegetable products of the Himalayas. Indian Journal of Microbiology.

[b0455] Tamang J.P., Tamang B., Schillinger U., Guigas C., Holzapfel W.H. (2009). Functional properties of lactic acid bacteria isolated from ethnic fermented vegetables of the Himalayas. International Journal Food Microbiology.

[b0460] Tamang J.P., Watanabe K., Holzapfel W.H. (2016). Review: Diversity of microorganisms in global fermented foods and beverages. Frontiers in Microbiology.

[b0465] Thakur K., Rajani C.S., Tomar S.K., Panmei A. (2016). Fermented bamboo shoots: A rich niche for beneficial microbes. Journal of Bacteriology and Mycology.

[b0470] Vilanova C., Porcar M. (2016). Are multi-omics enough?. Nature Microbiology.

[b0475] Voidarou C., Antoniadou M., Rozos G., Tzora A., Skoufos I., Varzakas T., Bezirtzoglou E. (2021). Fermentative Foods: Microbiology, Biochemistry, Potential Human Health Benefits and Public Health Issues. Foods.

[b0480] Wagner Anika Eva, Boesch-Saadatmandi Christine, Dose Janina, Schultheiss Gerhard, Rimbach Gerald (2012). Anti-inflammatory potential of allyl-isothiocyanate– role of Nrf2, NF- κB and microRNA-155. Journal of Cellular and Molecular Medicine.

[b0485] Waisundara V., Jayawardena N., Watawana M. (2016). Regulating Safety of Traditional and Ethnic Foods.

[b0490] Wan Yu-Jun, Shi Hui-Fang, Xu Rou, Yin Jun-Yi, Nie Shao-Ping, Xiong Tao, Xie Ming-Yong (2019). Origin of hypoglycemic benefits of probiotic fermented carrot pulp. Journal of Agriculture and Food Chemistry.

[b0495] Wang W., Xia W., Gao P., Xu Y., Jiang Q. (2017). Proteolysis during fermentation of Suanyu as a traditional fermented fish product of China. International Journal of Food Properties.

[b0505] Woo M., Kim M., Noh J.S., Song Y.O. (2017). Kimchi methanol extracts attenuate hepatic steatosis induced by high cholesterol diet in low-density lipoprotein receptor knockout mice through inhibition of endoplasmic reticulum stress. Journal of Functional Foods.

[b0510] Wu Z., Li J., Huang L., Zhang X. (2021). Basic pH reversed-phase liquid chromatography (bRPLC) in combination with tip-based strong cation exchange (SCX-Tip), ReST, an efficient approach for large-scale cross-linked peptide analysis. Analytica Chimica Acta.

[b0515] Yang Liang, Fan Wenlai, Xu Yan (2020). Metaproteomics insights into traditional fermented foods and beverages. Comprehensive reviews in Food Science and Food Safety.

[b0520] Yesiltas Betül, Gregersen Simon, Lægsgaard Linea, Brinch Maja L., Olsen Tobias H., Marcatili Paolo, García-Moreno Pedro J. (2021). Emulsifier peptides derived from seaweed, methanotrophic bacteria, and potato proteins identified by quantitative proteomics and bioinformatics. Food Chemistry.

[b0525] Zang J., Xu Y., Xia X., Regenstein J.M. (2019). Quality, functionality, and microbiology of fermented fish: A review. Critical Reviews in Food Science and Nutrition.

[b0530] Zhang Ping, Zhang Pengfei, Xie Mengxi, An Feiyu, Qiu Boshu, Wu Rina (2018). Metaproteomics of Microbiota in Naturally Fermented Soybean Paste. Da-jiang. *Journal of Food Science*.

[b0535] Zhao Shengming, Han Jinzhi, Bie Xiaomei, Lu Zhaoxin, Zhang Chong, Lv Fengxia (2016). Purification and Characterization of Plantaricin JLA-9: A Novel Bacteriocin against *Bacillus* spp. Produced by *Lactobacillus plantarum* JLA-9 from Suan-Tsai, a Traditional Chinese Fermented Cabbage. Journal of Agricultural and Food Chemistry.

